# Northwestern Dental Association

**Published:** 1885-01

**Authors:** S. J. Hill


### PROCEEDINGS.

### Northwestern Dental Association.

There having been no regular meeting held in July, a special meeting was called at Moorhead, Minn., Nov. 18, 1884, for the transaction of business.

The Secretary reported eight new names for membership, and the same were ordered to be added to the roll.

The financial report shows the Association out of debt, and a fair balance left in the treasury. Several interesting papers were read and discussed by the members present.

The main business of the Association was in regard to dental legislation for Dakota.

Drs. Louis Ottofy, of Ottofy, Dak.; S. J. Hill, of Fargo, Dak., and J. W. Cloes, of Jamestown, Dak., were appointed a committee on legislation, and plans were discussed and adopted looking to the introduction of a bill at the coming session of the Territorial Legislature, embodying the features of the law adopted by the National Association of Dental Examiners.

The co-operation of all dentists in the Territory is earnestly solicited, and it is thought the prospects for success are very fair.

Louis Ottofy, President, S. J. Hill, of Fargo, Secretary, were each re-elected.

We have now nineteen active members, eleven corresponding, and one honorary.

Fargo, Dak., was selected as the place for holding the next meeting on the fourth Tuesday of July, 1885.

The meeting adjourned at the close of the evening session, with the feeling that quite a step forward had been gained by the profession in this new country, and we hope soon to join the ranks of the older States in measures which will tend to elevate to a higher plane all who pursue our calling.

S. J. Hill, Secretary.

